# Complete genome of the mutualistic symbiont *Buchnera aphidicola* AIST from a Japanese strain of the pea aphid *Acyrthosiphon pisum*

**DOI:** 10.1128/mra.00973-24

**Published:** 2024-12-23

**Authors:** Masaki Mizutani, Ryuichi Koga, Takema Fukatsu, Shigeyuki Kakizawa

**Affiliations:** 1 Bioproduction Research Institute, National Institute of Advanced Industrial Science and Technology (AIST), Tsukuba, Japan; 2 Department of Biological Sciences, Graduate School of Science, University of Tokyo, Bunkyo-ku, Japan; 3 Graduate School of Life and Environmental Sciences, University of Tsukuba 13121 , Tsukuba, Japan; Queens College Department of Biology, Queens, New York, USA

**Keywords:** insect endosymbiont, genome, PCR-amplified fragment

## Abstract

The genome of *Buchnera aphidicola* National Institute of Advanced Industrial Science and Technology (AIST), an obligate bacterial endosymbiont from a Japanese strain of the pea aphid *Acyrthosiphon pisum*, was determined. The genome sequence provides valuable information for comparative and evolutionary aspects of the intimate insect-microbe mutualism.

## ANNOUNCEMENT

Mutualistic relationship with microbial symbiont is indispensable for growth and reproduction of diverse insect species, especially those that rely on nutritionally unbalanced diets like plant sap (1, 2). *Buchnera aphidicola* is a clade of essential symbiotic bacteria of aphids. These bacteria are known for supplying essential amino acids to compensate for protein-deficient plant sap diet of the insect host and being vertically transmitted to the next generation through ovarial passage (3–5). Despite the genome reduction, the symbiont retains the capability of synthesizing most essential amino acids while being devoid of most genes for synthesizing non-essential amino acids, which reflect the role of nutritional supplementation at the genomic level (5, 6).

Here, we resequenced the *Buchnera* genome from a Japanese aphid strain AIST, maintained in the laboratory since 1999 (7). DNA was extracted from aphids by using conventional proteinase K digestion and phenol extraction (8). Entire *Buchnera* genomic regions were amplified by long PCRs from extracted DNA and sequenced. In total, 55 primer sets were designed to amplify the entire 680 kbp *Buchnera* genome (GenBank accession number NC_002528). Each primer was 34–35 nucleotides (Table 1), and the resulting fragments ranged from 4 to 24 kbp. PCRs were performed using KOD-One polymerase (TOYOBO) according to the manufacturer’s instructions. All 55 fragments were successfully amplified and purified using a PCR Purification Kit (QIAGEN). For library construction, the purified PCR products were fragmented, tagged using an MGIEasy FS DNA Library Prep Set (MGI Tech), circulated using an MGIEasy Circularization Kit (MGI Tech), and rolling amplified using an DNBSEQ-G400RS High-throughput Sequencing Kit (MGI Tech). The constructed DNA nanoballs (DNBs) were sequenced using DNBSEQ-G400 (MGI Tech) platform in a 200 bp paired-end mode. Adapter sequences were trimmed using CLC Genomics Workbench 23.0.4 (QIAGEN). Default parameters were used for all software unless otherwise specified. The obtained 11,738,882 paired reads were mapped onto the reference genome (NC_002528), then the 11,473,424 mapped reads were applied to a *de novo* assembly using CLC Genomics Workbench. The contigs were manually assembled to construct a single circular genome with 3,582-fold coverage. For circularization, paired reads overlapping both contig ends were used, and the genome was rotated based on the reference genome. The final genome was polished by the read mapping using CLC Genomics Workbench and annotated using DFAST pipeline v.1.6.0 (https://dfast.ddbj.nig.ac.jp/).

**TABLE 1:** PCR primer pairs used in this study

Region	Amplified length (bp)	Forward primer name	Forward primer sequence (5'−3')	Reverse primer name	Reverse primer sequence (5'−3')
1–2	23,800	1-F	TTATCCACAGATTTGTTCTTTACTAATAATAATAG	2-R	TTTAATTAAAAGATCTTTCTTGGTATATAAGTCTT
3–4	23,800	3-F	GATATTTTGAGAAAAATAGTTTTGACTTATAAAGT	4-R	CAGGAGAAATGATCCGCGTCAATGATGGTCCTTTT
5	12,000	5-F	TCTCATTTTATATAAAATTTTTATTAAAATTTACA	5-R	CTACTTTGGAATTACCATTAGACTCTGCTACCTCT
6	12,000	6-F	TGCTTCTATTTGACGAATTCTCTCTCGAGTAACAT	6-R	AAATTTACTTCTGAAATTTCTATTATTTATAACGG
7	12,270	7-F2	CTGAAATCATAACATTACCTTTAATAATAAAGTTT	7-R2	AATAGAATTTTTTGTGTCGCTCGACATAAATGGTT
8	12,135	8-F	AACATAAAAATAATTTCAATAAAATTCATGAATCA	8-R2	TGAATATCAGATGTTAAAGTATTTTTTGAAAAACT
9–10	23,800	9-F	GGCATTGGGATTGTTTCTAGCATTGGTAATAATAA	10-R	TTGTAATTAATGGGAGAAAAATCCCAAGTAAACGA
11	12,000	11-F	TACTTTCGTTATACTCACATCATCAGTATTATTAT	11-R	AGTTAATATCATCAGGATGGTAATTCGGCGCACGA
12	12,000	12-F	TCCAGTAGGTTTTAAAAATGGAACTGACGGTAATA	12-R	ATCAGAAGTTTTCTCGTCCTATTCGTAACATTGTG
13	12,000	13-F	GAAATAGTCAGCAATGATATTATTTTTTTCAAGAA	13-R	AATTGGGATCAAGGTAGTTTTACTTTTAAAAAAAA
14	12,000	14-F	CCGTCTGGAGATAAATTTAATACAGTTTTAGGATC	14-R	ATATACGCTTCAGGACGAGGTGGACAACCAGGAAT
15	12,000	15-F	GGTACGCCATTTATAAAAATGGCGCCTGTTATTCA	15-R	CGAGAATGTATATCAGGTAACCATCCATGAAACGG
16	12,000	16-F	ATATTGCTATCTTCTATTTTGTTATTAGTTTTTAG	16-R	TATTCCATTGCAAATAGCAGAAATAGAAGATCCAT
17	12,000	17-F	CTGAAACTTCTTTTCTATATGCAATACCATATAAT	17-R	AATAAAGGGCATCGAGCTTACATTAGAATCTTTAC
18	12,000	18-F	TTAAAACTATTTTATTAAAAGTAAAGCCTTTTTTA	18-R	AATTTTTACATCTCCACTCGATAACATCCCACGCC
19	12,000	19-F	TTCGTGTTCGAGAATTGGGTCAAGAAATTACCAAG	19-R	AGATACAGTAGGAAATTGCCAAAAATTTGGCATTA
20	12,000	20-F	CTAAAAATCATAAAGATGGAGGTGATTTAGTTTAT	20-R	TTTATTAGGTGGTGTATCTTCTGAAAGAAAAATTT
21	12,100	21-F	CTTGAATACTTCCATCTTCACCACCTTTCCCATGT	21-R2	TGAAATTTTGAAAAAATTAGGACAAAATGGTCGAT
22	12,000	22-F	CATTCGGAAGCAGAAATTCCATAATTAGGATTCAT	22-R	GGAAAAACAGGAAGACATTAATATTTCCATATTTA
23	12,000	23-F	ATCTAACTTTTTTCGAACCTGATTTTACTCAGTTT	23-R	GTTTTTTGATCGCTTCTTTTGTTTGTATTAACAAA
24	12,000	24-F	ATTAACTAGTATCGTGCGTATTGCATTAAGAAAAA	24-R	ATTGGTATAACATGAATCATATTGTGACAAAAAAA
25	12,000	25-F	GATATATGGAAAATGTTGATATAAAGCATTGGCGA	25-R	CATTATTAAAACATTCAGATAAATTTGGACAATGA
26–1	7,239	26-F	ATTAAAAAAATTAAATATTATCAATATTAAAAATC	26-R7	TATATGCACAAGGATTTATTTATGTATTATCTCG
26–2	6,200	26-F2	TAAACTAATTTTATCTATTTCATTATAAAAAATCC	26-R2	ATACGTTTAAACATTTTTTTTGACATTCAGAGTAA
27	12,000	27-F	CTATTCCATTTGCATTAATGTTATATGGAGGTATG	27-R	ACAATATAATTCTTTAATACTACGTCAGATTTAAA
28	12,100	28-F2	GAGGTGGAGCATTTTCAAAAAATGCAATCGGATTA	28-R	TTTTGGTAGTGATACTGGAAACACAGAAAAGATAG
29	12,000	29-F	TTAAAGTTGGCAAGAAATCATCCCAATCACATTGA	29-R	GATCAAAAGCGTAATAATGTTGTTGTTTCACGTCG
30	12,000	30-F	TTCCAAGCCATATCAGTAATATGTAAAAGACCATC	30-R	AAGACTGCACTAATGACATCGAGGATTACATCTGG
31	12,000	31-F	AACTCGCCACGACATAAAAAACCTCGTTTACGTAA	31-R	TGGTCTCATTTTAATCCTTTAAAATATGATATGAA
32–1	8,900	32-F6	GTAATAAAAGAAACGAGTCAGTACAAAGTATATC	32-R5	TTAATTATTTTATCTTCTGTTTTAAAAGATTGAGC
32–2	6,200	32-F3	CCAATTATCTACATTAATTGGACGTCATGTGATGG	32-R3	TTGCTTAATTTCTTCTGTATGTTTTCATTAAAAAC
33	12,135	33-F2	AAAATAAAAAAATCTTATTAAAAAAGGATAAAAAA	33-R	TAATTGGAAAAAGCAATATATGATGACTGTTTCCA
34–1	6,235	34-F2	GAATCTATCAGGAGGTTTTATTTTTAAAATGTATG	34-R3	AATAAAAATAATAGCTTACGAATGAAATAATAAAA
34–2	6,100	34-F3	ATAAAAATATAACATAAATAGAAAAGTTATCAATT	34-R	TTTTATAAATATAATGTTTTAGGCCGAATTTATGT
35	12,000	35-F	TTTTTTTTAATTTTAACACAAAGAACCCAAAAAGA	35-R	AATAATCAATATATTTTAAATCCTATTAGTGATGA
36	12,000	36-F	TTTGATAGAGAACGAATATTATTAATTACCACTTG	36-R	ATGTACACCAGGAGGTGATGTTGAATATAAAATTT
37	12,000	37-F	CTTGACATATTGATCTTGAAAATGTTCTAATAACC	37-R	TTATTAAATGCGAATAAAATAAACGATCAAGATGT
38	12,000	38-F	GTGCTTTCTCCTCTTATTGCTCGTACTCCGATACC	38-R	GCAAATGATTAAAGGTGGAGGAGGTGCTTTAACAA
39	12,000	39-F	GATATTTTGGCGTTCCACCAATTTTTATCATTTCT	39-R	ACTAATTACATTTGGTCGATCTAAATTAACACCAA
40	12,000	40-F	ATATATTTTCACTTTGAAACCATGCTAGTTTCTTA	40-R	AGTCACTTTATCATTAGTCTTAATAGAACCTTGTT
41	12,000	41-F	CAGGGACAGATTATTTGAATATGCAAAATAACATG	41-R	CATAGGAAGACAATAATATTTTTCTTTTTTTGGAT
42	12,270	42-F2	TATTAATATTGATGGTTATTCTTTTAAAATTTCTA	42-R2	AATACTTGTATGATCAGTTCCAATCATACATATTA
43	12,000	43-F	AATGGCAAAAAAAACTATCAAAATTACAATACCTT	43-R	TTTTTATCAAGCTCTTTATATCGTACGTTTAATAA
44	12,000	44-F	TGAATACAGCTTAAAATAGCTATATCAATCGATGG	44-R	TTGTTTTGTGTATTGTTTATATAAATTTTTTATTT
45	12,000	45-F	AAAACATCACGGAAAAAAAAACGATTCAAACGGAC	45-R	ATTATGCTATCAATATTAATTGTATTGTGAAAGTA
46	12,000	46-F	ACCAACTATTCAATATTGCAGTGTTTGGAGCTAAC	46-R	TCAAACAGATAATACATTTTTTTTTGATTGGTCTC
47	12,000	47-F	AGTAAATTAAGTTTGATTGTTTATTATTTTAAAAA	47-R	TTTGGTCCTGTTACACGTGAATTAAGAACAGAAAA
48	12,000	48-F	CTTGATTTAAAGATAAAATATTTTTAATAATACCT	48-R	CGACTAAAATTCAATTGAAAAAAAACGCAAAAGAA
49	12,000	49-F	ACCATTAATTAGTGTACCTTTATAATGAACAGTGA	49-R	GATGAATATTGAAAAAGAACGTGGAATTTCTATAA
50	12,270	50-F3	AGTATTCATTTAATGAACCATCTAAAAAACTATCT	50-R	TAATTTTTTTATACATTAGATGATAATGAAAAAAA
51	12,000	51-F	AAAAACAATTTTTTATACTTTTGAAAAGAAAAATC	51-R	AAAATTAATAGATCCCATCTCTGCGAGTCGAATTC
52	12,000	52-F	GTTCAATTTTATTGTTTAACCATTCTTTATTCGGA	52-R	AAATTAATATAAACACCTTCTGTTTCCATAAGTGC
53	12,000	53-F	ATTTTATTTATAGCATCTGGAGCATTTCAAACATC	53-R	TTACCGACAACAAAAATCCTGCATCCGAATAACCA
54	12,000	54-F	TTTAAAATTTGTCTAATTGAATGTTATAAAAGAGT	54-R	GCGGCATTACATCTAACAATATAACTTTATTTTTA
55	3,681	55-F	TAAAGAGGTGATCATGGTAGGAGGGTCTACTCGTA	55-R	ACATATAAAAACCTTTTAAAAATTTAAAAATATTC

The complete circular genome of *B. aphidicola* AIST was 641,802 bp in size with 26.3% G + C content. It encoded putative 582 protein-coding sequences, 32 transfer RNAs, and a single copy of 16S/23S/5S ribosomal RNA operon. The amino acid synthesis pathways reported in *Buchnera* strains from *Acyrthosiphon pisum* (5, 8) were identified in the results of the present genome analysis. These results are consistent with prior studies highlighting the nutritional roles of *Buchnera* (5). Our new symbiont genome data will contribute to comparative symbiont genomics that provide insight into how sophisticated insect-microbe mutualisms have evolved and diversified. Additionally, the genome sequencing adopted in this study, in which the entire genomic regions were amplified by PCR before sequencing, will be useful for sequencing a genome from a tiny amount of DNA.

## Data Availability

The sequence data have been deposited to DDBJ under the accession numbers AP036055 for assembly and DRA019179 for raw reads.
